# Orientation-tuned surround-suppression is strongest within perceived 3D surfaces

**DOI:** 10.1167/jov.25.4.17

**Published:** 2025-04-29

**Authors:** Jessy K. Possel, Pieter R. Roelfsema, Matthew W. Self

**Affiliations:** 1 Department of Vision and Cognition, Netherlands Institute for Neuroscience, Amsterdam, the Netherlands; 2 Department of Integrative Neurophysiology, VU University, Amsterdam, the Netherlands; 3 Department of Neurosurgery, Amsterdam University Medical Centre, Amsterdam, the Netherlands; 4 Laboratory of Visual Brain Therapy, Sorbonne Université, Institut National de la Santé et de la Recherche Médicale, Centre National de la Recherche Scientifique, Institut de la Vision, Paris, France; 5 School of Psychology and Neuroscience, University of Glasgow, Glasgow, Scotland

**Keywords:** depth perception, perceptual organization, surround suppression, contrast perception, contextual processing

## Abstract

Our perception of contrast is strongly affected by contextual factors. A well-known example is that of orientation-tuned surround suppression (OTSS). Surrounds have more suppressive effects on the perceived contrast of the center when they have the same orientation. This phenomenon has been linked to horizontal interactions within the early visual cortex. Here we show that the strength of suppression is modulated strongly by the perceived three-dimensional structure of the scene. Placing the surround at a different retinal disparity, and therefore perceived depth, decreased OTSS, regardless of whether the surround was placed behind or in front of the center. The difference in disparity was, however, not the key determinant of the strength of OTSS. Suppressive interactions were strongest when the surround formed a three-dimensional surface that was continuous with the center and weaker when the surround seemed to be part of a separate surface, even when the surround seemed to be closer to the center in perceived depth. The results suggest that visual features that are perceived to form part of the same surface can engage in stronger suppressive interactions than those perceived to lie on different surfaces. This raises questions about how the underlying neural interactions become restricted to surfaces and support the view that top-down information about perceptual organization can gate interactions occurring at lower levels in the visual system.

## Introduction

Throughout the visual processing hierarchy, neural circuits compare local visual information with the surrounding context. These circuits suppress regions of the visual scene that are structured uniformly and act to enhance regions of spatially changing visual information (Angelucci et al., 2017). The function of this process is to enhance contrast at multiple levels, from enhancing luminance contrast to enhancing the contrast between different visual features. In this way, the visual system enhances the representation of objects, which typically differ along several feature dimensions from the background. A classic example of such a process is surround suppression. The response of a neuron in visual cortex is suppressed by the addition of visual information to the area surrounding its receptive field, although the surround does not evoke a response if it is presented in isolation (Hubel & Wiesel, 1962; Knierim & van Essen, 1992). The amount of surround suppression depends on the orientation of the surround; an iso-oriented surround generally produces more suppression than a cross-oriented surround, a phenomenon known as orientation-tuned surround suppression (OTSS) (Blakemore & Tobin, 1972; Knierim & van Essen, 1992; Levitt & Lund, 1997; Maffei & Fiorentini, 1976; Nelson & Frost, 1978; Self et al., 2014; Shushruth et al., 2012; Sillito, Grieve, Jones, Cudeiro, & Davis, 1995; Trott & Born, 2015). Mechanistically, OTSS is thought to arise through horizontal interactions between orientation-tuned cells and local interneurons in the visual cortex (Adesnik, Bruns, Taniguchi, Huang, & Scanziani, 2012; Angelucci et al., 2017; Angelucci & Bressloff, 2006; Bosking, Zhang, Schofield, & Fitzpatrick, 1997; Stettler, Das, Bennett, & Gilbert, 2002) and through feedback connections from higher cortical areas, although the exact circuitry has not been established. A simple mechanism in which orientation-tuned pyramidal cells send horizontal connections to inhibitory interneurons connected to similarly tuned neurons could, in principle, underlie OTSS.

However, such a hardwired circuit would predict that cells should always experience maximum suppression when the surround is at the preferred orientation of the cell. This is not what is observed; OTSS is strongest when the surround and centre have the same orientation, even if that orientation drives the cell suboptimally (Shushruth et al., 2012). This finding suggests that more flexible circuits underlie OTSS. Feedback input from higher visual areas could fulfil this function and, indeed. if neuronal activity in higher visual areas is blocked, the suppression of iso-oriented backgrounds is weaker (Kirchberger et al., 2021; Nassi, Lomber, & Born, 2013; Nurminen, Merlin, Bijanzadeh, Federer, & Angelucci, 2018; Vangeneugden et al., 2019).

**Figure 1.**
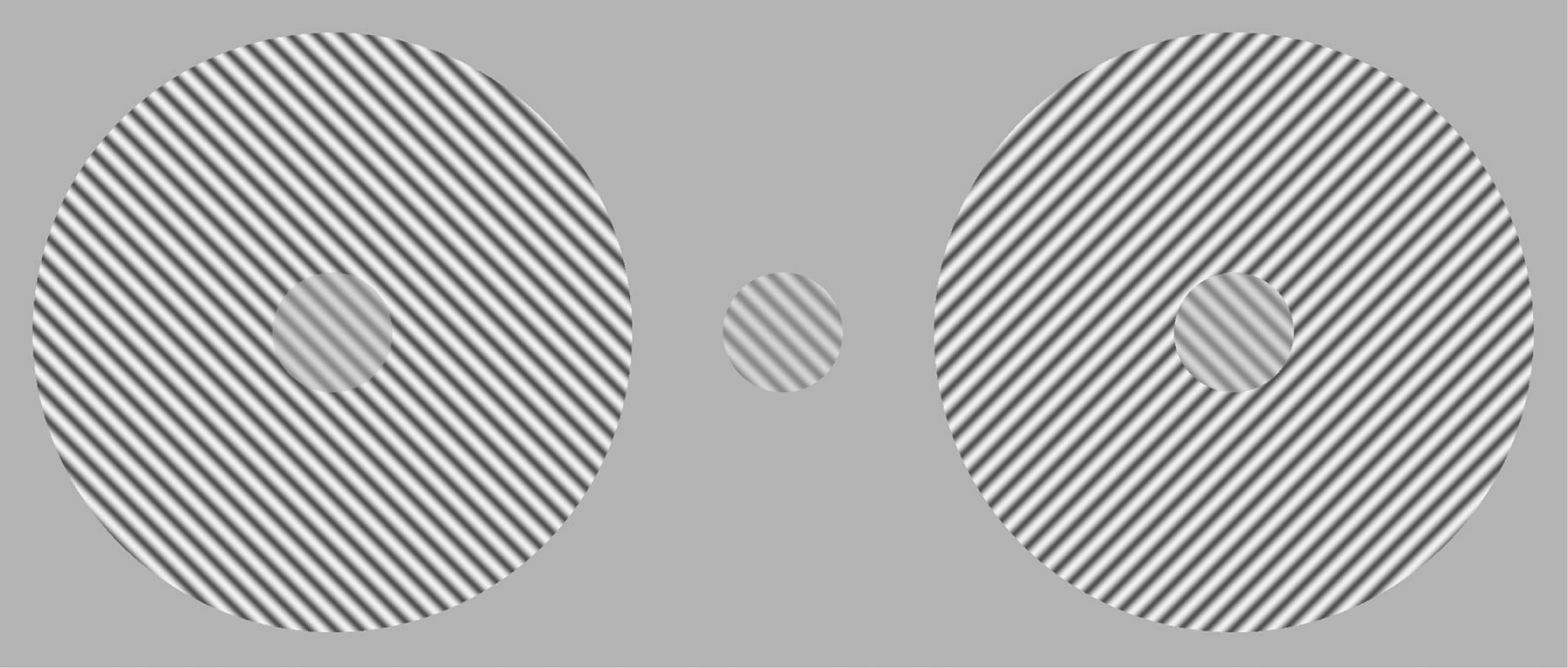
Iso-oriented surrounds reduce the perceived contrast of central targets. The central grating has the same physical contrast in all three images.

Perceptually, OTSS results in lower perceived contrast when a target is embedded in an iso-oriented context compared with a cross-oriented context (Figure 1) (Cannon & Fullenkamp, 1991). Contrast perception is not only influenced by the orientation of the surround, but also by other factors such as the contrast of the surround (Chubb, Sperling, & Solomon, 1989; Levitt & Lund, 1997) and whether the object is perceived as a figure or as part of the background (Self, Mookhoek, Tjalma, & Roelfsema, 2015). Figure–ground segregation is typically tested with stimuli that closely resemble those used to test OTSS: a central target surrounded by a texture of the orthogonal orientation. In most studies, OTSS and figure–ground segregation are confounded and both effects could explain the observed changes in perceived contrast. In general, the perceived contrast of an image region depends on the three-dimensional (3D) interpretation of the scene (Lotto & Purves, 2001). The visual scene is segmented into different 3D surfaces and objects and contextual phenomena depend on this interpretation (Lotto & Purves, 2001; Nakayama, Shimojo, & Silverman, 1989). In this study, we therefore set out to determine whether the effect of OTSS on perceived contrast is hardwired into the visual system or whether it can be modified by changing the perceptual organization of the scene. We manipulated figure–ground relationships between the surround and center by placing the surround at different depths relative to the center. In this way, we were able to dissociate figure–ground segregation and OTSS and examine their effects on perceived contrast separately. The results indicate a clear rule—OTSS is strongest when the surround and center are perceived as a single surface and weaker when the surround belongs to a different surface. The results support the idea that the strength of OTSS is not determined fully by local features, but can be modulated by the brain's 3D perceptual model of the scene.

## General methods

This study was reviewed and received ethics clearance through the ethics committee at the University of Amsterdam. Informed consent was obtained from all individual participants in the study. Participants were informed that they could withdraw from the study at any time without penalty. The raw data from each experiment are available publicly via the Open Science Framework via the following link: https://osf.io/tuvmb/ with DOI: 10.17605/OSF.IO/TUVMB. The code used to generate the stimuli and analyze the data will be shared by the lead contact upon request.

In all experiments, we estimated the perceived contrast of a sine wave test grating by asking observers to compare its contrast with a reference grating that varied in contrast on different trials. Reference and test gratings were presented in different temporal intervals and the perceived contrast difference between the reference and the test grating was estimated using a two-alternative forced choice with an opt out approach, as described elsewhere in this article. We first describe the general approach common to all experiments before describing each experiment individually.

### Stimuli

All stimuli were created in MATLAB (The MathWorks Inc., Natick, MA) using Cogent Graphics (developed by John Romaya at the LON at the Wellcome Department of Imaging Neuroscience) and displayed on a linearized cathode-ray tube monitor (full screen 36.5° × 27.4°; 1,600 × 1,200 pixels, 85-Hz refresh rate). The stimuli were presented to both eyes using a stereoscope (Sokkia, Japan) at a viewing distance of 54 cm. Eight square random dot patterns (1.1° per side) were used as a fixation lock, which surrounded the stimuli and were present at all times. The stimuli consisted of a test grating and a reference grating, with an orientation of 45° and a diameter of 2°. The test grating was always presented at 30% Michelson contrast and was surrounded by either a 45° or 135° oriented annulus (iso-oriented or cross-oriented, respectively) with a diameter of 8° and 80% contrast. The reference grating was presented with varying contrasts per trial. All stimuli had a spatial frequency of 2 cyc/deg and were drawn on a gray (38.76 cd/m^2^) background.

### Experimental procedures

Observers were seated in a dimly lit room. We first verified that the participants were able to detect objects embedded in random dot stereograms and that they could perform basic depth from disparity discriminations. We then showed the observers examples of gratings at different contrasts and explained the concept of judging whether gratings are higher or lower in contrast than a reference. Observers then performed a training task to get accustomed to making contrast judgments and using the do not know option (as described elsewhere in this article). In these training tasks we used a two-alternative forced choice with opt-out design (2-AFC-DK) (García-Pérez & Alcalá-Quintana, 2012; Self et al., 2015) to test the ability of the participant to report their contrast perception veridically. Participants were presented sequentially with two sine wave gratings. Both gratings were presented without any surround and at zero disparity. The reference grating was presented at one of nine contrasts drawn from 21% to 39% in steps of 2.25%. The test grating was presented at either 27.75% or 32.25% contrast. The order of the gratings was chosen pseudo-randomly. Participants had to indicate whether the second grating was higher or lower in contrast than the first grating by pressing the up/down arrows on the keyboard with their right hand. In addition, the observers could respond do not know (DK) by pressing the D key with their left hand. Observers were encouraged to use DK option if the gratings seemed to be the same contrast or if they were unsure what the correct answer was. Participants performed 432 trials (12 repeats for each contrast/presentation order/reference grating contrast). The data were analyzed immediately to estimate the participant's perceived contrast of the two test gratings as described elsewhere in this article, if these were within a reasonable margin of error from the true physical contrast (±1%); then, the participant progressed through to the main experiment. If not, the participant repeated the training procedure. All participants successfully completed the training within two sessions.

For the main experiment, observers performed multiple sessions. Before the start of each session, we first ensured that participants were fusing the two images correctly and the point of zero horizontal disparity was measured using nonius lines. In each experiment we estimated the perceived contrast of different test gratings using the 2-AFC-DK as described below. The order of presentation of the test and reference grating and the different experimental conditions were chosen pseudo-randomly and were balanced within observers.

### Estimation of perceived contrast differences

We used a 2-AFC-DK design in which participants indicated on each trial which grating was higher in contrast or indicated that they did not know. Responses in the 2-AFC-DK design can be modelled using the difference-with-indecision model described by García-Pérez and Alcalá-Quintana (2012). In this variant of signal detection theory, the representation of the difference in contrast between the test and reference grating for a given contrast of the reference grating is assumed to be normally distributed (with parameters: mean [μ] and standard deviation [σ]). If the experimental manipulation affects the perceived contrast of the test grating, this would shift the mean of this distribution away from zero. On individual trials, the participants are assumed to respond according to two internal criteria: if the signal difference falls below criterion δ1, then they respond with “reference grating lower” and if it falls above δ2 they respond with “reference grating higher.” For signal differences that fall in between δ1 and δ2, they respond DK. Unbiased participants should place these criteria symmetrically around zero; response biases are, therefore, captured by shifts in these criteria. The model simultaneously fits four parameters (μ, σ, δ1, and δ2) to generate six psychometric functions (3 response options [reference higher, reference lower, DK] × 2 presentation orders [reference 1st/2nd]). The mean parameter (μ) describes the effects of the surround on the perceived contrast of the test grating; positive (negative) values indicate that the experimental manipulation increases (decreases) the perceived contrast of the test grating. The parameter μ coincides with the maximum probability of responding DK when averaged across the conditions in which the test grating was presented first or second. At this contrast, the participants are maximally uncertain about their response and we use this parameter as an estimate of the difference in perceived contrast between the test and reference grating. This approach also controls for biases that participants may have in responding to a particular spatial location or temporal interval (García-Pérez & Alcalá-Quintana, 2012).

To fit the difference-with-indecision model to each observer's data we first calculated the fraction of the trials on which the observer responded that the reference grating was higher in contrast, lower in contrast or that they were uncertain (DK response). These fractions were computed separately for the trials on which the reference grating was presented first or second. We then fitted the difference-with-indecision model using maximum likelihood estimation. The mean parameter (μ) derived from the fit was taken as the difference in perceived contrast between the test and reference gratings and was used as the statistic for across-observer analyses. We also examined whether the other parameters of the model were affected by the experimental manipulations. The effects on steepness (σ) and bias (δ1 and δ2) were largely nonsignificant and inconsistent across experiments (Supplementary Figure S1), and for this reason we focused our analysis of the mean parameter, μ.

## Results

### Experiment 1

**Figure 2.**
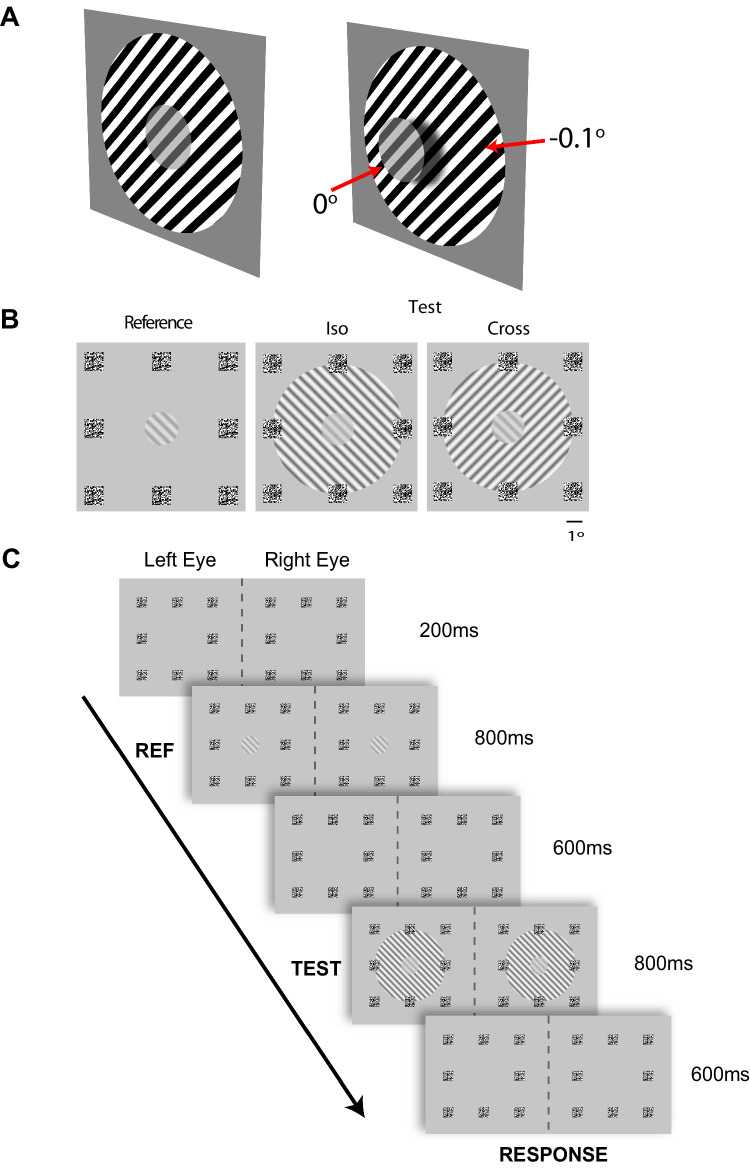
Design of Experiment 1. (**A**) Schematic representation of the depth manipulation for the Iso condition. (Left) In the flat condition, a central test grating of 30% contrast was surrounded by an 80% contrast surround grating, which appeared in the same depth plane. (Right) In the depth condition, the surround was presented with a negative disparity of 0.1°, making it appear at a greater distance from the observer than the central grating (shadows added for emphasis here). (**B**) Screen shots showing the stimuli. The eight random-dot patterns were always present on the screen at zero disparity to aid binocular fusion and fixation at the zero disparity depth plane. (**C**) The time-course of a single trial. The trial began with the fusion markers. Then either the reference (REF) or test condition was presented. The reference grating was shown in both eyes at zero disparity. After a 600-ms interval, the other condition was shown. At the end of the trial, the participant indicated which interval contained the highest contrast or indicated they did not know.

In this experiment, we determined whether OTSS is sensitive to binocular disparity. Observers were asked to compare the contrast of an isolated reference grating, which could seem to be at different contrasts, with the contrast of a test grating which was surrounded by a contextual grating (Figure 2A). The experiment consisted of a 2 × 2 design with the factors being 1) the relative orientation of the surround and center grating and 2) the relative depth of the center and the surround. The central grating was always presented with an orientation of 135° (i.e., leftward tilting). The surround was either iso-oriented (i.e., also 135°, and with the same phase as the central grating) or cross-oriented (45°) relative to the center. The central grating was always presented at zero disparity (the fixation plane). The surround grating was presented either at zero disparity (the Flat condition) or with a negative disparity of −0.1° so that it seemed to be behind the plane of fixation, with the central grating appearing relatively closer to the observer (the Depth condition). The test grating was always presented in isolation and at zero disparity (Figure 2B).

#### Procedure

Nine observers participated in this experiment with age ranging from 20 to 26 years (5 males; average age, 22.4 years). All subjects had normal or corrected-to-normal vision and were right handed. All observers, except one, were naïve to the aims of the experiment. The observers performed the 2-AFC-DK contrast discrimination paradigm as described in the General methods. The reference gratings were presented at 30% Michelson contrast. On the basis of pilot experiments, the contrast of the reference grating was chosen to range from 18% to 34% in steps of 2%; additionally, we added two easy conditions where the reference contrast was 14% or 38%, resulting in a total of 11 different reference contrast values. The reference contrasts were presented in a balanced pseudo-random order. The surround grating had a fixed contrast of 80%.

Each session consisted of 440 trials and was repeated 8 times, yielding a total of 3,520 trials per participant. The trial started with a fixation period (200-ms duration) during which participants were instructed to fixate on the red fixation dot (eye movements were not measured). Then, observers were presented with either the test grating or the reference grating for 800 ms. After a 600-ms inter-stimulus interval, the second grating was presented for a further 800 ms. The inter-stimulus interval screen was then shown for a minimum of 600 ms, after which the participant was allowed to respond. Figure 2C illustrates the experimental procedure.

#### Results

**Figure 3.**
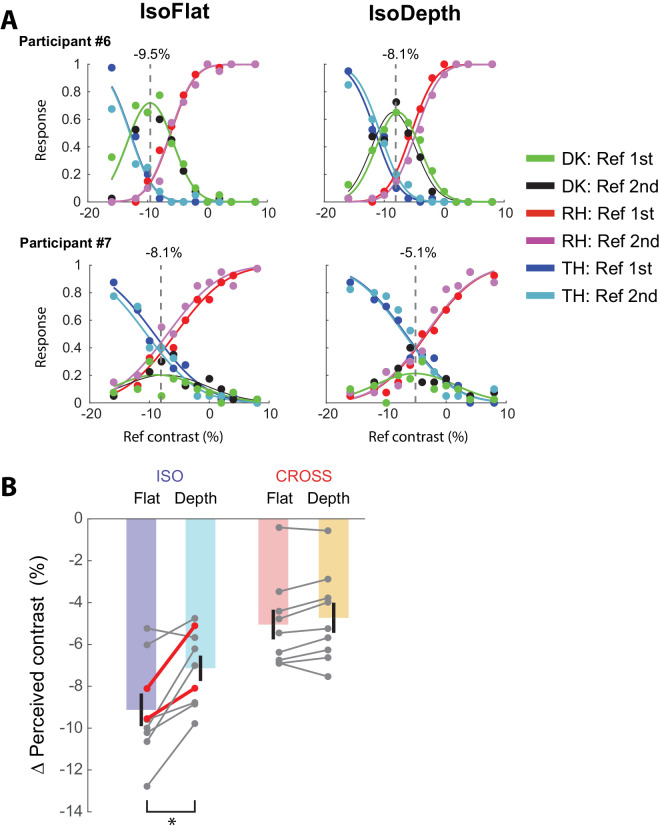
Placing the surround at a different depth decreases iso-orientation surround suppression. (**A**) The psychometric functions of two example participants in Experiment 1 for the conditions IsoFlat, and IsoDepth. The *x* axis shows the difference in physical contrast between the reference grating and the test grating, which had a physical contrast of 30%. The *y* axis shows the proportion of trials on which the participant chose the reference grating as being higher in contrast (RH, red and magenta curves), the test grating as higher in contrast (TH, blue and cyan curves) or indicated do not know (DK, black and green). The red, blue, and green points come from the condition when the reference grating was presented in the first interval, the magenta, cyan, and black points when the reference grating was presented in the second interval. The curves show psychometric functions fitted for the different orderings and represent estimates that are uninfluenced by decisional bias. The dashed line indicates the estimate of perceived contrast of the test grating relative to the reference grating, taken as the maximum of the averaged psychometric functions for the uncertain responses. (**B**) Estimated ∆ perceived contrast values for the four conditions. All eight participants (gray markers) perceived the contrast of the test grating on an iso-oriented surround to be lower than on a cross-oriented surround. The addition of depth increased perceived contrast in the Iso condition, but not in the Cross condition, *p = 0.013, Bonferroni corrected post hoc *t* test. The red lines/markers indicate the two example participants in the panel above. The error bars represent the standard error of the mean (%).

The difference-with-indecision model (Alcalá-Quintana, García-Pérez, & Miguel, 2013) provided a good fit to the data of eight observers (Figure 3A). The proportion of the trials on which the observers used the uncertain response ranged from 2.8% to 24.1% with an average of 12.2%. Figure 3A shows example fits for two participants in the IsoFlat (iso-oriented surround, no depth difference) and IsoDepth (iso-oriented surround, different depth) conditions. The perceived contrast difference between the reference and test gratings was estimated as the peak of the fitted the DK condition. The perceived contrast of the test grating was lower than that for the reference grating in all conditions (Figure 3B). This was expected, because the test grating was surrounded by a high-contrast grating, which it is known to reduce perceived contrast (Chubb et al., 1989), whereas the reference grating was presented without the surround. On top of this general surround effect on perceived contrast, we observed a significant main effect of the orientation of the surround on perceived contrast, repeated measures 2 × 2 analysis of variance: F_1,7_ = 15.8; *p* = 0.004. Iso-oriented surrounds were more suppressive than cross-oriented surrounds (−8.13% vs −4.89%). Furthermore, there was a significant main effect of the relative depth of the surround compared with the center, F_1,7_ = 18.4, *p* = 0.003, with flat surrounds being more suppressive than surrounds at a different depth (−7.1% vs −5.9%). Most important for this experiment, there was a significant interaction between the orientation of the surround and the relative depth of the surround, F_1,7_ = 12.95, *p* = 0.007. The interaction was driven by a significant increase in perceived contrast when the iso-oriented surround was moved to a different depth, post hoc *t* test, *p* = 0.013, Bonferroni corrected. There was no significant effect of moving the cross-oriented surround to a different depth, post hoc *t* test, *p* = 0.29, Bonferroni corrected.

#### Discussion

Experiment 1 showed that OTSS is indeed sensitive to binocular disparity. However, this is only true for iso-oriented stimuli. Placing the surround at a different depth decreased the amount of surround suppression in the Iso condition, but had no influence in the Cross condition. A plausible explanation for these results is that iso-suppression might be sensitive to disparity, with the strongest suppression occurring for centers and surrounds at the same disparity. One previous study (Gheorghiu, Kingdom, Thai, & Sampasivam, 2009), which examined the suppression of shape adaptation by textured surrounds, suggested that iso-suppression might be weaker when the surround is placed at a different disparity. An alternative hypothesis is that suppression depends on the figure–ground status of the center. In the IsoFlat condition, the center grating does not segregate from the background strongly, because the background and the center share the same orientation and phase. However, in the IsoDepth condition, the central grating can be perceived as a figure in front of the background with a different depth. In the Cross condition, the central grating is always perceived as a figure owing to the difference in orientation, and depth might have no influence on the perceived contrast. This interpretation is in line with previous work showing that figures are perceived as being greater in contrast than backgrounds (Self et al., 2015). Experiment 1 did not allow us to dissociate between an effect of figure–ground segregation and an effect of the different depth planes. This will be the topic for Experiment 2.

There was an additional effect of depth in the Iso conditions, because depth-by-disparity also introduced a phase difference between the central grating and the surround. Phase is an important cue for segmentation of surfaces and phase offsets have been shown to greatly reduce the strength of surround suppression on the perceived contrast of sine-wave gratings (Olzak & Laurinen, 1999; although see McCourt, 2005; Xing & Heeger, 2001). Similarly, at the neural level, the strength of surround suppression has shown to be sensitive to phase offsets (Li, Jiang, Wang, Xie, & Yao, 2018; Xu, Shen, & Li, 2005). Phase differences could therefore also account for the difference in surround suppression between the IsoFlat and the IsoDepth conditions. In Experiment 2, we removed any contribution of phase differences by jittering the relative phase of the center and surround across trials.

### Experiment 2

**Figure 4.**
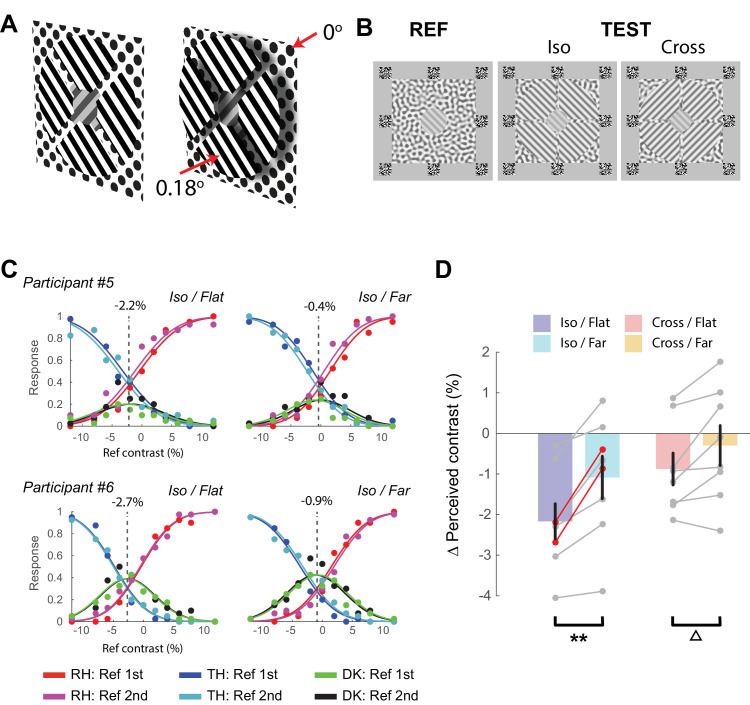
Moving the surround toward the observer causes less suppression. (**A**) Schematic of the experimental design. The reference grating was surrounded by four wedges which could either be in the same depth plane (left) or placed closer to the observer (right). (**B**) Screen shots of stimuli. The test grating was surrounded by non-oriented texture to reduce non-orientation specific suppression effects. (**C**) Example fits of the difference-with-indecision model to the data of two participants in the IsoFlat and IsoFar conditions. Conventions as in Figure 3A. (**D**) ∆ Perceived contrast of the reference grating for the four conditions. **p < 0.01. ∆, *p* = 0.06. DK, do not know; RH, reference grating higher in contrast; TH, test grating higher in contrast.

In Experiment 1, we demonstrated that the suppressive effects of an iso-oriented surround could be reduced by placing the surround at a different depth relative to the center. It is unclear, however, whether this was due to a true depth-induced release from suppression or due to the change in the figure–ground relationship between the center and the surround. Furthermore, the disparity could have created a phase offset between the center and surround, causing a partial release of the suppression. To address these issues, we designed Experiment 2 in which the surround was placed nearer to the observer so that the central grating was perceived as part of the background. If the changes in perceived contrast in Experiment 1 were caused by the figural status of the central grating, then the perceived contrast should be lower in the presence of a depth difference. In addition, we added a random phase offset to the surround grating so that there was no consistent phase relationship when depth was added to the stimulus. We also added a high-contrast non-oriented surround to the reference grating. The non-oriented surround resulted in test and reference gratings that were well-matched for non-orientation tuned surround suppression (Figure 4A).

#### Procedure

Nine subjects participated in this experiment with ages ranging from 20 to 24 years (4 males; average age, 22 years). All subjects had normal or corrected to normal vision and were right handed. Aside from one participant, all participants were naïve to the aim of the experiment. Six of the participants had participated in the previous experiment. The experimental design was identical to Experiment 1 consisting of a 2 × 2 factorial design with one factor being the relative orientation of the surround and center (cross or iso) and the other factor being the relative depth of the surround (near or flat).

Initial pilot experiments indicated that the depth percept was not convincing when a simple annulus was used as the surround grating. To create a convincing depth percept, we created slots in the annulus (0.33° in width), dividing it into four wedges (Figures 4A and 4B). Furthermore, we slightly increased the size of the central grating to 2.5° so that the surround annulus did not abut the central grating directly. These two changes produced an immediate and stable perception of depth in the Near conditions. To create the Near conditions, we added uncrossed disparity to the stimulus in the right eye (0.18°) so that the wedges appeared nearer to the observer (Figure 4A). The phase of the central and surround gratings was chosen randomly from a uniform distribution on each trial. In all cases the reference grating was surrounded by a non-oriented control texture at 80% contrast that was presented at zero disparity. The texture had the same spatial frequency as the gratings (2 cyc/deg.) and was created by filtering Gaussian noise (regenerated on each trial) through a series of oriented Gabor filters (18 orientations equally spaced between 0° and 180°). Each session consisted of 440 trials and was repeated 8 times, yielding in a total of 3,520 trials per participant.

#### Results

We analyzed the data by fitting the difference-with-indecision model as described elsewhere in this article to estimate the relative perceived contrast of the test grating in each of the four conditions. The proportion of the trials in which the participants used the uncertain response ranged from 2.6% to 45.5% with an average of 21.4%. The difference-with-indecision model provided an excellent fit for all participants (see examples in Figure 4C). Figure 4D shows the relative perceived contrast for the four conditions. There was a significant main effect of orientation on perceived contrast, F_1,8_ = 69.3, *p* =3 × 10^−5^, as iso-oriented surrounds produced more perceptual suppression than cross-oriented surrounds (−1.5% vs −0.5%). In addition, there was a significant main effect of depth, F_1,8_ = 17.7, *p* = 0.003, as flat surrounds were more suppressive than near surrounds (−1.4% vs −0.6%). There was also a significant interaction between orientation and depth (F_1,8_ = 17.8, p = 0.003). The interaction was driven by a greater increase in perceived contrast when the surround was nearer for iso-oriented than cross-oriented surrounds. As in Experiment 1, the effect of moving the surround to a different depth produced a significant release of suppression for the iso-oriented surrounds, post hoc *t* test, *p* = 0.004, Bonferroni corrected. For the cross-oriented surrounds there was a non-significant trend in the same direction, post hoc *t* test, *p* = 0.06, Bonferroni corrected.

#### Discussion

As in Experiment 1, we observed a release from OTSS when the surround was moved to a different depth than the center. This result is not explained by figure–ground segregation because figures tend have be perceived at a higher contrast (Self et al., 2015). Furthermore, the experiment also controlled for the phase relationship between the center and surround as the relative phase was chosen randomly on each trial. The result therefore indicates that iso-orientation suppression is sensitive to differences in binocular disparity.

In both experiments, the reduction in perceived contrast produced by an iso-oriented surround grating was reduced by moving the surround to a different depth. The result could be explained by two different underlying mechanisms. The first possibility, which we refer to as the disparity tuned hypothesis, proposes that the suppression is strongest when the center and surround have the same retinal disparity and is weaker when they have different retinal disparities. This hypothesis suggests an underlying neural architecture whereby disparity-tuned neurons tend to suppress neurons with a similar disparity and orientation preference. Such a model would explain the observed pattern of a release from suppression when the surround is placed at a different disparity to the center.

An alternative explanation is that suppression is not tuned for disparity but instead depends on the perceived 3D structure of the scene. Suppression might be strongest when the center and surround are part of the same surface, and suppression might be released when the center and surround are perceived to be part of different surfaces. We, therefore, refer to this hypothesis as the surface hypothesis. This hypothesis suggests a more high-level form of suppression that depends on the perceptual interpretation of the scene.

### Experiment 3

**Figure 5.**
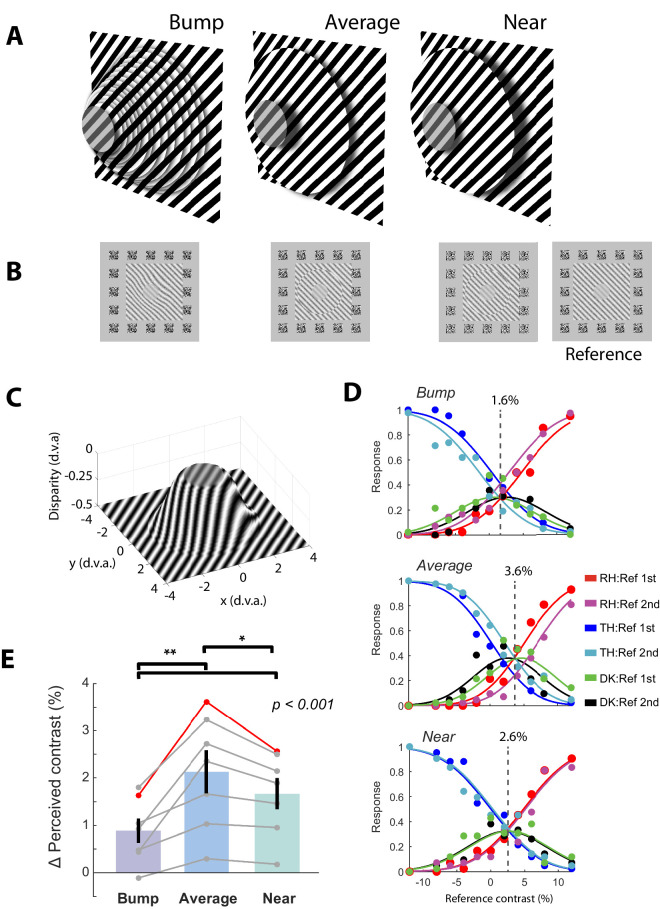
Design and results of Experiment 3. (**A**) Schematic of the stimuli used for experiment 3. The bump stimulus was composed of concentric rings at closely spaced disparities to create the perception of a smooth bump (see **C**). The average stimulus contained one annulus at the average disparity of the rings used for the bump stimulus. The near stimulus was an annulus placed at a closer disparity than the average. (**B**) Screen shots of the right eye stimulus for each condition, plus the reference stimulus for which the contrasts of the central grating varied. (**C**) The actual disparities used for the bump, the *z* axis shows disparities in degrees of visual angle (d.v.a.). (**D**) Example fits of the difference-with-indecision model for one participant in the three conditions. Conventions as in Figure 4A. (**E**) ∆Perceived contrast values for each stimulus relative to the zero-disparity reference stimulus. Error bars are 1 standard error of the mean. Gray lines show individual subjects. The *p* values come from Bonferroni-corrected post hoc *t* tests, *p < 0.05; **p = 0.01. DK, do not know; RH, reference grating higher in contrast; TH, test grating higher in contrast.

This experiment was designed to discriminate between the disparity-tuned and surface-based hypotheses. As discussed elsewhere in this article, the disparity-tuned model suggests that the strength of suppression depends on the similarity in disparity between the center and surround, with maximal suppression if disparity is the same. The surface-based model proposes instead that suppression is strongest when the center and surround form part of the same perceptual surface and should be weaker when they are perceived as separate surfaces. To distinguish between these hypotheses, we attempted to dissociate differences in disparity from the perception of surfaces. We designed two complementary stimuli containing disparity differences between the center and surround (Figures 5A–C). The first stimulus was a smooth 3D bump which that pointed toward the observer. The center seemed to be part of the same surface as the surround, but the surround had a different average disparity. The second stimulus was perceived as containing two separate discs, the center disc at zero disparity and the surround disc at the average disparity of the surround in the Bump condition (the Average condition). We also added a third (Near) condition to control for the possibility that surround suppression is dominated by the near surround where the surround had a smaller disparity in the Bump condition.

#### Procedure

Seven subjects participated in this experiment with age ranging from 19 to 27 years (3 males; average age, 22.6 years). All subjects had normal or corrected-to-normal vision and were right-handed. Aside from one participant, all participants were naïve to the aim of the experiment. None of the participants participated in the previous experiments. This experiment had three conditions: Bump, Average, and Near as described elsewhere in this article. The Bump stimulus was constructed using a cumulative Gaussian function defined in radial coordinates centered on the center of the bump. The disparity of the bump was then defined by:

Disparity=1-CDF(x,μ,σ),

where *x* was the radial coordinate system in pixels, μ was the mean of the cumulative Gaussian, set to produce a flat central region at the top of the bump of 2.26° diameter, and σ was the steepness of the bump, set to 0.5°. The resulting function was normalized so that the center was at 0 disparity and the base of the bump at a disparity of −0.5°. The function was then rounded to the nearest pixel value to quantize the curve (Figure 5C). The bump was constructed as a series of 21 circles, one per pixel of quantized disparity. The circles contained a grating stimulus as used in the previous experiments. A random noise pattern was overlaid on the grating to strengthen the depth percept. The final result was perceived by observers as a smooth bump with a flat top. To construct the Average condition we used a single disc set to the average disparity of the bump (−10 pixels or 0.22°). The Near condition also contained a single disc set to a disparity of −5 pixels (0.11°); this is the average disparity of the bump stimulus in a ring of 1° width surrounding the center stimulus. As in the previous experiments, disparity was only added to the stimulus in the right eye. In all cases, the reference grating was surrounded by an iso-oriented control texture at 80% contrast and presented at zero disparity. Each session consisted of 396 trials and was repeated 7 times, yielding in a total of 2,772 trials per participant.

#### Results

We analyzed the data by fitting the difference-with-indecision model as described elsewhere in this article to estimate the relative perceived contrast of the reference grating in each of the three conditions. The proportion of the trials in which participants used the uncertain response ranged from 14.9% to 48.9% with an average of 29.4%. The difference-with-indecision model provided a good fit for all participants (see example in Figure 5D).

Figure 5E shows the relative perceived contrast for the three conditions. Note that the perceived contrast difference values were positive in this experiment; the reference stimulus consisted of a grating surrounded by an iso-oriented, flat surround, whereas the test conditions all contained surrounds at different disparities which were less suppressive. There was a significant main effect of condition on perceived contrast, F_2,12_ = 20.6, *p* = 0.0001, because both the Average and Near conditions resulted in greater perceived contrast than the Bump condition (2.1% and 1.7% vs 0.89%), both *p* = 0.01, Bonferroni-corrected post hoc *t* test. In addition, there was a significant effect of disparity, *p* = 0.04, Bonferroni-corrected post hoc *t* test, because the Near surround produced significantly lower perceived contrast than the Average surround.

#### Discussion

The disparity-tuned suppression hypothesis proposes that the suppression is strongest when the center and the surround have the same disparity and is weaker when they have different retinal disparities. However, in Experiment 3 we have shown that suppression was stronger in the Bump condition than in the Average condition, even though in both conditions the surround had the same average retinal disparity. The difference between these conditions is that the center seems to be a part of the same surface as the surround in the Bump condition, whereas it is perceptually segregated from the surround in the Average condition. This finding suggests that surround suppression is strongest within perceptual surfaces. One concern with this conclusion might be that the surround in the Bump condition contains a range of disparities and, because disparity increases with distance from the center, the surround regions closest to the center (the near surround) have a more similar disparity to the center than the surround in the Average condition. If the surround-suppression effect is dominated by the near surround, this could explain the difference in perceptual suppression between these two conditions. To address this concern, we included the Near condition in which the surround was placed at a closer average disparity to the center. The level of suppression in the Near condition was again significantly weaker than in the Bump condition, supporting the perceptual surface hypothesis and speaking against the disparity hypothesis. We note, however, that the similarity in disparity between the center and surround did play a role in the strength of suppression, because suppression was stronger in the Near condition than in the Average condition. We cannot rule out, therefore, that iso-orientation surround suppression is tuned for disparity. It would be revealing to investigate the effect of the disparity of the surround disc at a finer range of disparities in further experiments.

## General discussion

In this study, we found that the influence of the surround on the perceived contrast of the center was modulated by grouping factors which altered the 3D perception of the scene and grouped the center and surround into one continuous surface. We used binocular disparity to place the surround at different depths relative to the center. In each of the experiments, the surround caused the strongest contrast suppression when it was at the same perceived depth as the center. These findings are in accordance with the general principle that suppressive interactions are strongest if the features of the center and surround are similar (Cannon & Fullenkamp, 1991; Ejima & Takahashi, 1985; Olzak & Laurinen, 1999; Sato, Motoyoshi, & Sato, 2012). However, this principle cannot explain the results of Experiment 3, in which the surround with the most similar feature values to the center (the Near condition) was less suppressive than the Bump condition, in which the center and surround were part of the same 3D surface. Instead, we propose that image regions, which are perceived to be part of the same surface, engage in stronger suppressive interactions than regions that belong to different surfaces. This proposal is related closely to the work of Nakayama and his colleagues, who have shown that visual features that belong to the same surface are grouped together in apparent motion paradigms and that surface-based operations can explain performance in face recognition and visual search tasks (He & Nakayama, 1994; Nakayama et al., 1989). Our results indicate that there are also suppressive interactions between nearby surfaces; in Experiment 3 the Near surround was still more suppressive than the Average surround, which was perceived to be further away from the center. There may be a continuum of suppressive interactions, where features that are closer together in 3D space can interact more strongly than those perceived to be further apart. This could be viewed as a 3D extension of the known relationship between retinotopic distance and surround suppression strength (Angelucci et al., 2017; Angelucci & Bressloff, 2006; Angelucci & Bullier, 2003; Bair, Cavanaugh, & Movshon, 2003; Cannon & Fullenkamp, 1991).

Surround suppression is a fundamental computational principle of sensory systems that acts to suppress the neuronal activity in uniform regions of sensory feature maps and enhance the activity in regions with feature contrast. In visual orientation maps, regions with high orientation contrast are more likely to be located at the boundaries between objects and backgrounds. By enhancing the representations of these regions, the visual system enhances the representation of objects. Surround suppression is not a single process, but occurs at multiple levels of visual processing and spatial scales. A large fraction of surround suppression is not tuned to orientation: high-contrast surrounds suppress the perceived contrast of the center, irrespective of their orientation. This phenomenon has been well described in the Chubb illusion (Chubb et al., 1989). The strength of the Chubb illusion is strongly modulated by whether the surround and center are perceived to be part of the same object or different objects (Lotto & Purves, 2001). We reasoned that similar high-level grouping mechanisms may also affect the strength of the orientation-tuned component of surround suppression. Surrounds have more suppressive effects on perceived contrast if they have the same orientation as the center (Cannon & Fullenkamp, 1991; Solomon, Sperling, & Chubb, 1993). A simple model to explain such effects is that orientation tuned cells in early visual areas send projections that have suppressive effects on cells tuned to the same orientation. However, such a simple connectivity scheme cannot explain the full phenomenology of OTSS, and there are many suggestions from the electrophysiology literature that surround suppression is not hardwired into the horizontal connectivity between cells. For example, if surround suppression depended on horizontal projections, then the latency of surround suppression should increase as the surround is moved further away from the center, because horizontal connections are unmyelinated and slowly conducting. However, this relationship was not found in a study in the macaque visual cortex (Bair et al., 2003), where the latency of surround suppression was similar across a range of distances. Furthermore, the horizontal model predicts that the most inhibitory surround orientation should be the same as the preferred orientation of the cell. However, studies in the macaque cortex have shown that the most inhibitory surround orientation is not constant, but depends on the orientation of the center: the responses to off-preferred orientations, which still activate the cell, are most strongly suppressed by an off-preferred surround (Shushruth et al., 2012). To explain these findings, a more scale-invariant and flexible form of suppression is required, leading to the proposal that feedback projections may strongly contribute to surround suppression.

It is likely that the strength of suppression is modulated by feedback connections from higher visual areas as cooling studies (Nassi et al., 2013) and optogenetic studies (Nurminen et al., 2018; Vangeneugden et al., 2019) have shown that reducing the strength of feedback projections to V1 reduces surround suppression. Feedback projections also contain information about scene segmentation (Kirchberger et al., 2021; Kirchberger, Mukherjee, Self, & Roelfsema, 2023), which could in principle gate suppressive interactions between segregated surfaces. Alternatively, many of our results would be explained by a disparity-selective suppression mechanism, that is, suppressive interactions between cells tuned for the same retinal disparity. It is unknown whether such suppressive interactions exist in the visual cortex, although recent theories of surround suppression do suggest a role for inhibitory horizontal projections between cells with similar tuning preferences (Adesnik et al., 2012). This study proposed that horizontal projections from excitatory cells target a subclass of interneurons that express somatostatin (SST^+^ cells). SST^+^ cells are not suppressed by surrounding stimuli and if their activity is silenced optogenetically; this reduces the amount of surround suppression observed in nearby pyramidal cells (Adesnik et al., 2012). Horizonal connections onto SST^+^ cells with the same orientation preference could explain the orientation tuning of surround suppression, which is also observed in mice (Self et al., 2014), given that horizontal connections tend to connect regions of similar orientation preference (Bosking et al., 1997) and that SST^+^ cells are tuned for orientation (Ma et al., 2010). To explain our data, the horizontal connections would also have to be tuned for disparity; that is, cells that have similar disparity preferences would be more likely to suppress one another. Unfortunately, to our knowledge, nothing is known about the projections of disparity tuned neurons in visual cortex, so that it is difficult to assess how biologically plausible this model is.

Ultimately, disparity selective suppression cannot explain the results of experiment 3 in which the Bump stimulus produced the most suppression despite having a similar average disparity value as the other conditions. A model in which suppressive interactions are gated by the same mechanisms that segregate perceptual surfaces would be consistent with our results. The exact process by which the brain's internal model generates the perception of 3D structures is unknown, but it is likely that higher visual areas resolve ambiguities in the feedforward visual input and send information about the likely perceptual explanations back to early visual areas to alter activity patterns there (Roelfsema & de Lange, 2016). It may, therefore, be possible that an early suppressive effect due to local orientation information becomes overwritten once the scene is fully interpreted more fully. We have provided neural evidence for such an effect in a previous study, in which an early neural representation of likely proto-objects in a scene, based on local analysis, becomes overwritten once a global analysis of the scene is completed (Self et al., 2019). It is of interest to relate our findings to studies that examined the influence of colinear line elements on neuronal activity and contrast perception (Kapadia, Ito, Gilbert, & Westheimer, 1995; Li, Piëch, & Gilbert, 2006; Polat & Sagi, 1993, Polat & Sagi, 1994). These flanking line elements can have excitatory and suppressive (Pooresmaeili, Herrero, Self, Roelfsema, & Thiele, 2010) effects on neuronal activity and they can facilitate or inhibit the detection of low-contrast contour elements, depending on the density of image elements in the display and the distance between the image elements (Polat, Mizobe, Pettet, Kasamatsu, & Norcia, 1998). Future studies could examine whether the effects also depend on the perceived 3D structure and compare them with the present results. Our findings have important implications for the interpretation of both electrophysiological and perceptual studies of surround suppression that use bottom-up or local connectivity schemes to explain their findings. Our results indicate that the perception of contrast is complex, ultimately being determined by the brain's internal model of its visual environment. Classical contrast gain models (e.g., Carandini & Heeger, 1994), in which the perception of contrast is determined by local and contextual patterns of contrast and visual features, suffice in simple situations but fail in more naturalistic settings, where the global 3D perceptual organization of the visual scene influence perceived contrast. Unravelling how inferences about visual structure affect processing throughout the visual system remains an important goal toward understanding our perception.

## Supplementary Material

Supplement 1
