# Supplementary material for: Orientation-tuned surround-suppression is strongest within perceived 3D surfaces
Source: J Vis. 2025 Apr 29;25(4):17. doi: 10.1167/jov.25.4.17 (PMC12045118; doi:10.1167/jov.25.4.17)
Supplement: Supplement 1 [file jovi-25-4-17_s001.pdf]

Supplementary Figures

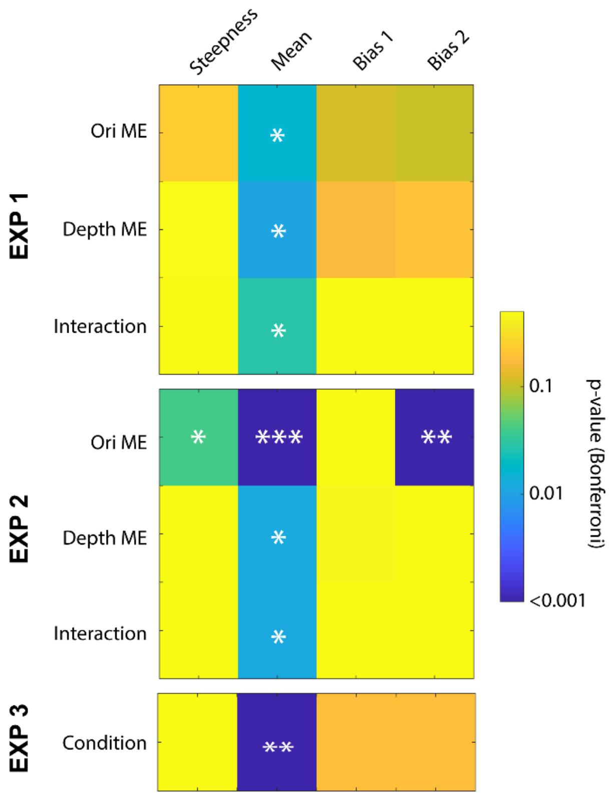

Supplementary Figure 1. Statistics from all three experiments on the four fitted parameters of the difference-with-indecision model. The p-values come from repeated measures ANOVAs and have been corrected for multiple comparisons using the Bonferroni method. Only the 'Mean' (μ) parameter was significantly modulated across all experiments. ME = main effect. *: $p < 0.05$, **: $p<0.01$, ***: $p<0.001$.
